# Minimally Invasive Replacement of a Missing Lateral Incisor With a Zirconia One-Retainer Bridge Using a Fully Digital Workflow: A Case Report

**DOI:** 10.7759/cureus.98149

**Published:** 2025-11-30

**Authors:** Wijdane Houta, Layla Assila, Amal El Yamani

**Affiliations:** 1 Department of Prosthodontics, Faculty of Dental Medicine, Mohammed V University, Rabat, MAR

**Keywords:** anterior tooth replacement, cantilever bridge, case report, digital dentistry, minimally invasive prosthodontics, resin-bonded fixed dental prosthesis, zirconia

## Abstract

Replacing a missing anterior tooth in young patients presents both esthetic and functional challenges, particularly when skeletal growth is not yet complete. Among conservative treatment options, zirconia cantilever resin-bonded fixed dental prostheses (RBFDPs) have demonstrated excellent outcomes with minimal biological cost. This case report describes the rehabilitation of a 20-year-old female patient who was missing the maxillary left lateral incisor, using a layered zirconia cantilever RBFDP fabricated through a fully digital workflow. The procedure involved intraoral scanning, computer-aided design, and computer-aided manufacturing (CAD/CAM) techniques. Minimal palatal enamel preparation was performed on the abutment tooth, incorporating macro-retentive features to improve bonding. The prosthesis was cemented with a dual-cure resin cement following a strict zirconia surface-treatment protocol. At the 12-month follow-up, the restoration demonstrated excellent esthetic integration, functional stability, and high patient satisfaction. This case highlights the effectiveness and minimal invasiveness of zirconia cantilever RBFDPs for anterior single-tooth replacement in carefully selected patients and emphasizes the value of digital workflows in enhancing precision and clinical predictability.

## Introduction

Tooth loss in the anterior esthetic zone presents a major clinical challenge for prosthodontists. Given its direct impact on function, appearance, and psychosocial well-being, prompt and well-planned intervention is essential. Several prosthetic options exist for replacing a single missing anterior tooth, including dental implants, resin-bonded bridges, and conventional fixed dental prostheses [1]. Among conservative solutions, cantilever resin-bonded fixed dental prostheses (RBFDPs), also referred to as single-retainer or one-wing bridges, are particularly notable. Their design requires only minimal preparation of the abutment tooth, maximizing preservation of enamel and sound hard tissues. This approach is especially suitable for young patients, offering a minimally invasive and reversible solution that preserves future treatment options while optimizing clinical effectiveness and patient comfort [2,3]. In the anterior region, cantilever designs have shown superior outcomes compared with two-retainer RBFDPs, demonstrating higher success rates and enhanced clinical performance [4,5]. Additionally, to avoid infra-occlusion associated with ankylosed implants, cantilever RBFDPs serve as an excellent provisional solution for patients with incomplete skeletal development, allowing implant therapy to be postponed until growth completion [6]. All-ceramic cantilever bridges, whether fabricated from glass-infiltrated alumina or zirconia, have demonstrated high reliability, durability, and esthetic performance. In particular, zirconia-based RBFDPs exhibit a 10-year survival rate of 98.2% and a success rate of 92.0% [7].

This case report aims to illustrate the use of a single-retainer zirconia cantilever bridge to replace a missing incisor using a fully digital workflow.

## Case presentation

Diagnosis and assessment

**Figure 1:**
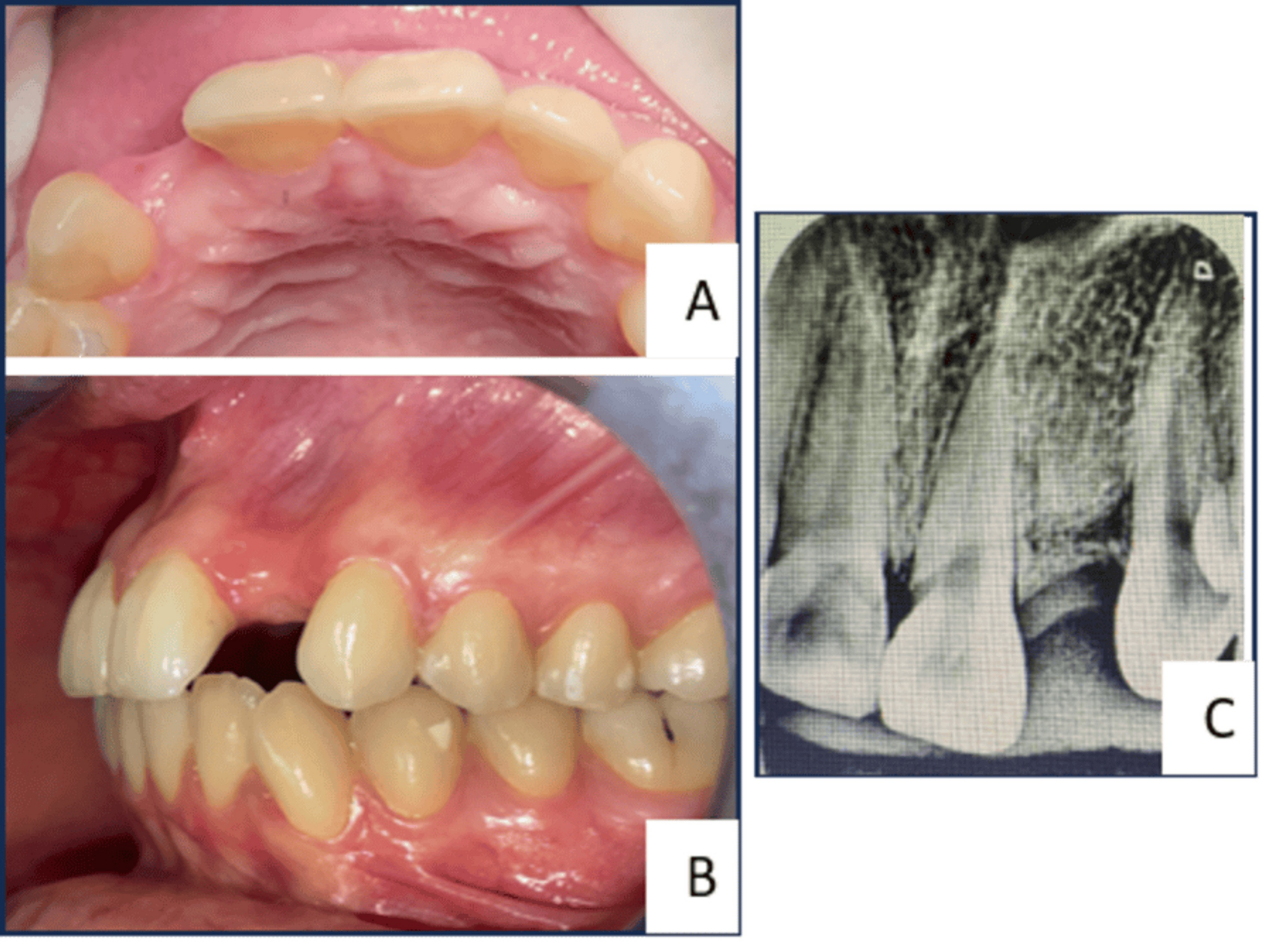
Initial clinical situation. A-B: Occlusal and lateral intraoral views showing absence of the maxillary left lateral incisor. C: Retro-alveolar pre-operative radiograph demonstrating moderate ridge resorption.

Clinical examination revealed the absence of the maxillary left lateral incisor. Oral hygiene was acceptable. Radiographic assessment showed moderate ridge resorption (Figure 1).

Abutment-tooth selection

Abutment selection was based on well-established clinical criteria. The maxillary left central incisor was selected instead of the canine due to its favorable alignment, angulation, pulp vitality, and healthy periodontium. Bone level, crown-to-root ratio, and root morphology were all favorable. The palatal enamel offered an ideal bonding substrate, and the proximal surface allowed a larger, more robust connector. Functionally, the load axis was closer to that of the missing lateral incisor. Occlusal contacts typically located on the canine cingulum could interfere with opposing canine guidance, potentially requiring excessive palatal reduction. The central incisor therefore represented the most appropriate abutment.

Occlusal and esthetic considerations

**Figure 2:**
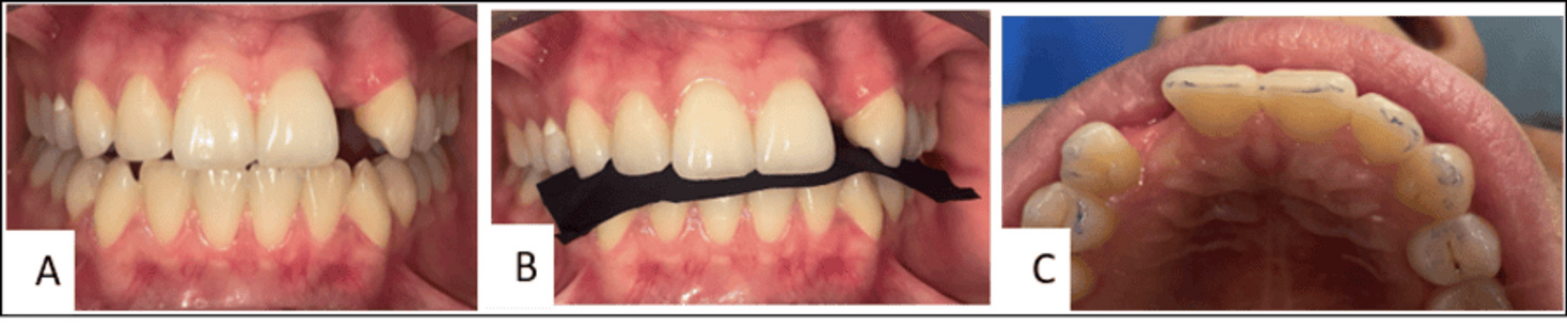
Occlusal contact evaluation. A-B: Use of articulating paper during static and dynamic mandibular movements. C: Articulating-paper markings revealing occlusal contact surfaces.

Occlusal contacts were evaluated using articulating paper in static and dynamic movements. Adequate interocclusal space for the retainer and pontic was confirmed. Incisal-edge anatomy was assessed to preserve natural translucency and guide margin design. Minimally invasive palatal preparation was planned, confined to enamel to provide a uniform bonding surface (Figure 2).

Therapeutic procedures

**Figure 3:**
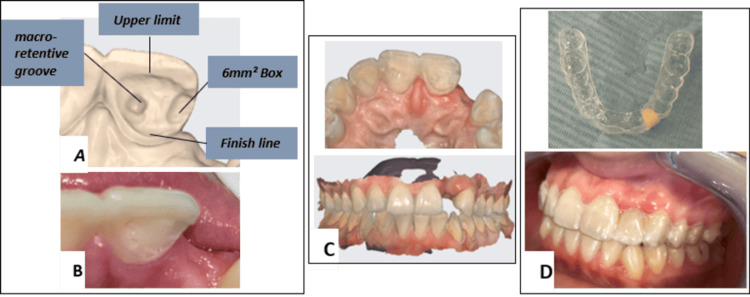
A: Abutment-tooth preparation (finish line, superior limit, distal box, central macro-retentive groove). B: Clinical view of the preparation. C: Digital impression obtained with an intraoral scanner. D: Temporization using an integrated temporary crown on a splint framework.

Palatal preparation of the maxillary left central incisor included a 0.5-mm deep finish line and a superior finish line 2 mm apical to the incisal edge. A distal proximal box (3 × 2 mm) and a 0.6-mm macro-retentive central groove were prepared (Figures 3A-3B).

A digital impression was obtained using an intraoral scanner. The maxillary arch, mandibular arch, and occlusion were scanned, and the STL files were transferred to computer-aided design (CAD) software (Figure 3C).

During temporization, a tooth-supported fixed provisional restoration integrated into a splint framework was fabricated to shape the ideal emergence profile through a non-surgical approach (Figure 3D).

CAD/CAM fabrication

**Figure 4:**
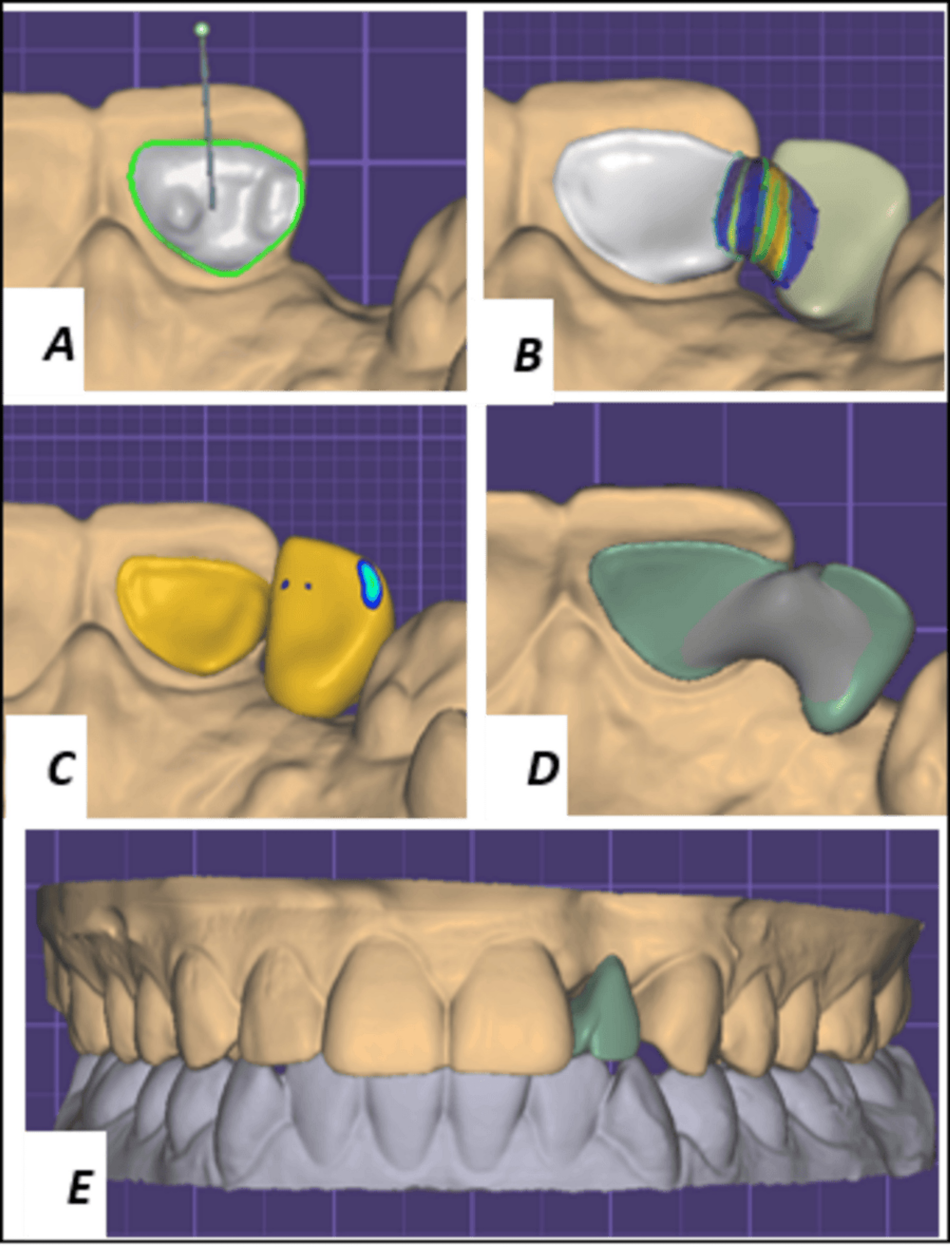
Computer-aided design procedures. A: Finish line of the preparation (green) and insertion axis. B: Pontic and connector design. C-D: Digital design of the pontic and connector in palatal view. E: Digital design of the pontic in buccal view.

**Figure 5:**
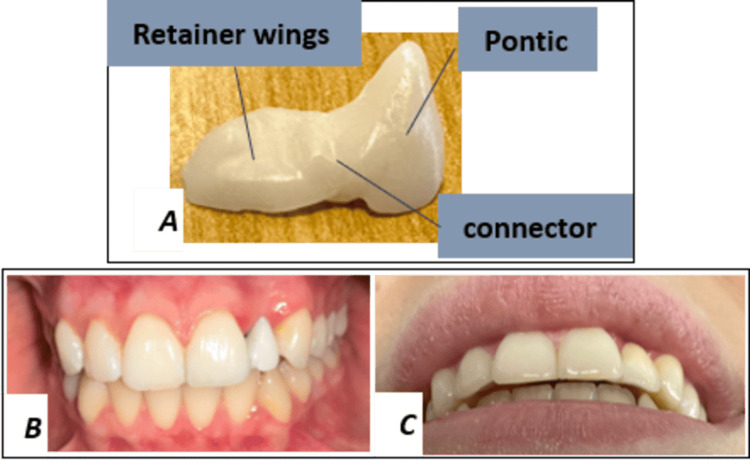
A: Zirconia framework fabricated from 3Y-TZP zirconia. B: Clinical try-in of the zirconia framework. C: Final try-in of the cantilever bridge after veneering ceramic layering.

The CAD phase involved automated margin detection and insertion-axis selection. Connector dimensions were designed to provide a minimum cross-section of 9 mm² to ensure adequate strength (Figure 4). The design was then transferred to computer-aided manufacturing (CAM) software and milled from 3Y-TZP zirconia using a five-axis milling unit (Figure 5A). A clinical try-in confirmed marginal fit and space for veneering ceramic (Figure 5B). The framework was then layered with an appropriately shaded veneering ceramic. A final try-in was performed before glazing (Figure 5C).

Bonding protocol

**Figure 6:**
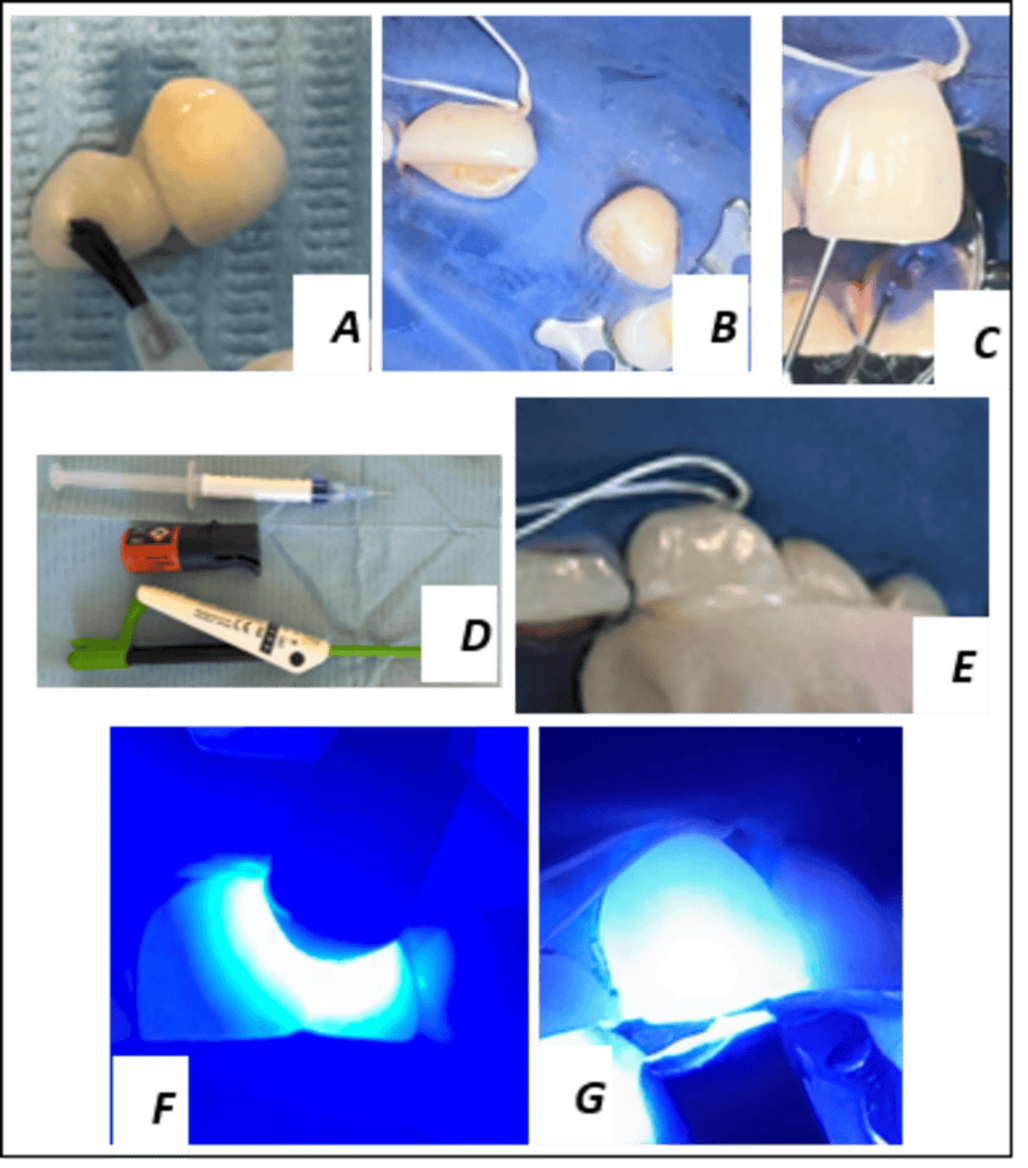
Assembly procedure. A: Internal surface treatment of the prosthesis. B: Dental isolation. C: Etchant application. D-E: Bonding with adhesive resin cement. F-G: Photopolymerization.

At the time of delivery, the prosthesis was cemented following the bonding protocol described below (Figure 6). The internal zirconia surface was conditioned with tribochemical silica coating: sandblasting with 50-µm alumina at 2 bar, silane application for three minutes, cleaning, and application of a ceramic primer containing 10-MDP (Figure 6A). Enamel surfaces were isolated with a rubber dam, etched with 37% phosphoric acid, rinsed, dried, and primed with a 10-MDP adhesive (Figures 6B-6C). The restoration was bonded using a dual-cure resin cement (Figures 6D-6E). Excess cement was removed, and polymerization was performed for 20 seconds per surface (Figures 6F-6G).

Occlusal verification and final outcome

After cementation, occlusion was carefully verified using articulating paper in both centric and excursive movements. The cantilever restoration was fully integrated into the patient’s static and dynamic occlusion, with no interference or overload on the pontic or connector. These clinical checks ensured proper occlusal harmony and long-term stability of the restoration.

**Figure 7:**
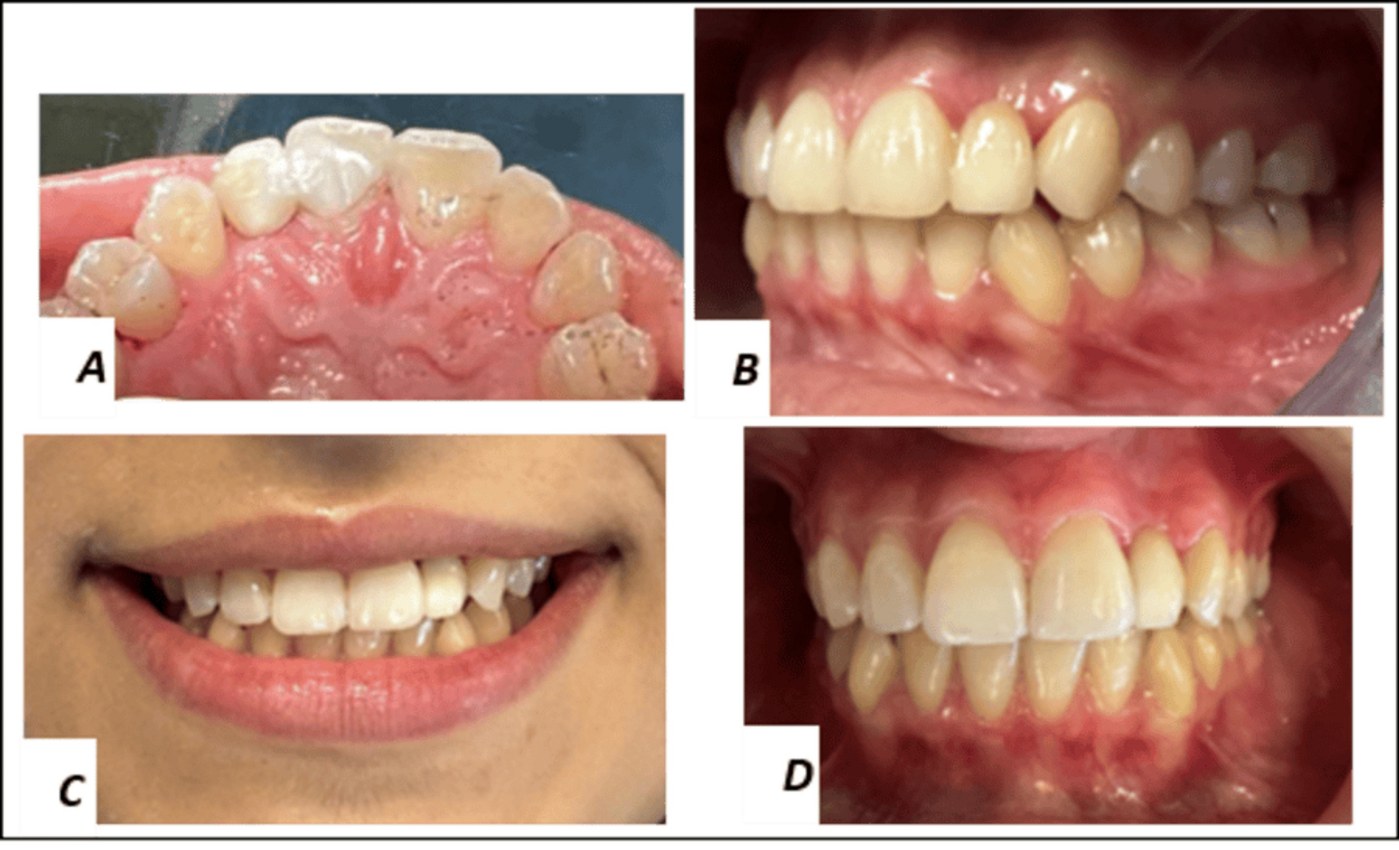
A-B: Postoperative clinical views. C-D: At the 12-month follow-up, the restoration showed excellent biological and esthetic integration.

The prosthesis also demonstrated excellent esthetic integration, harmonizing with the patient’s natural dentition. Proper alignment, contour, and morphology were achieved, confirming the success of the treatment (Figures 7A-7B). The patient expressed high satisfaction with both the esthetic outcome and functional performance of the restoration.

Follow‑up

At the 6- and 12-month recalls, the patient reported no discomfort. Esthetic and functional outcomes remained stable, reflecting the patient’s compliance (Figures 7C-7D).

## Discussion

Anterior tooth replacement remains a significant challenge in prosthodontics, particularly when a single edentulous space is involved. Multiple treatment approaches exist; however, the cantilever (single-retainer) all-ceramic resin-bonded fixed dental prosthesis (RBFDP) has emerged as a highly conservative and biologically favorable option for anterior tooth replacement [4,8-10].

The cantilever configuration preserves enamel, maintains pulpal vitality, and minimizes the risks of postoperative sensitivity and secondary caries, representing a clear biological advantage over more invasive fixed options [2,7,8]. In addition, single-retainer zirconia RBFDPs consistently demonstrate superior medium-term outcomes compared with two-retainer designs. Their success is largely attributed to the elimination of inter-abutment shear forces, one of the main causes of debonding in double-retainer RBFDPs, thereby improving long-term stability in the anterior esthetic zone [4,5,7,9,10].

Compared with metal-ceramic alternatives, all-ceramic cantilever RBFDPs exhibit higher survival rates and more favorable esthetic outcomes, reinforcing their reliability in anterior tooth replacement [10,11]. Zirconia remains the material of choice because of its high mechanical strength, fracture resistance, and ability to blend harmoniously with the natural dentition. Biomechanical analyses also demonstrate that restorations incorporating a 3-mm incisal overlap have the highest fatigue resistance, outperforming lithium disilicate and E-glass fiber-reinforced systems [9,12]. Long-term studies support this evidence, with Kern and colleagues reporting survival rates of 100% at three to six years and 98.2% at ten years, with a 92.0% success rate [13].

Other forms of zirconia, such as 5% yttria-stabilized tetragonal zirconia polycrystal (Y-TZP), have also shown clinical promise. These restorations exhibit a three-year survival rate of 82.7% and a six-year failure-free rate of 91.1%, increasing to 95.2% when excluding debonding events, highlighting that even in cases of debonding, restorations can remain functionally stable [1,14].

Recent advancements in digital technology have further strengthened the reliability of zirconia cantilever RBFDPs. Three-dimensional (3D)-printed zirconia crowns offer excellent esthetic results, while milled zirconia has shown slightly superior marginal accuracy. Notably, 3D-printed veneers exhibit greater precision in axial and incisal areas, particularly in restorations with complex morphology [15].

Fully digital workflows, combining intraoral scanning, CAD design, and CAM fabrication, have significantly enhanced precision, reduced laboratory steps, and minimized clinical errors. Fixed prostheses fabricated from digital impressions show improved marginal adaptation compared with those derived from conventional impressions, contributing to better biological and mechanical performance. From the patient’s perspective, digital workflows reduce chairside time, enhance comfort, and provide a more predictable, satisfactory experience [16,17].

## Conclusions

Cantilever zirconia RBFDPs provide a conservative, reliable, and highly esthetic solution for replacement of a single anterior tooth, particularly in young patients with incomplete skeletal development. This case demonstrates that combining minimally invasive tooth preparation with a fully digital workflow ensures precise fit, optimal esthetics, and functional stability while effectively preserving abutment tooth structure. At the 12-month follow-up, the findings support the clinical potential of this approach, demonstrating stable esthetic and functional outcomes. The long-term success of zirconia cantilever RBFDPs depends on careful patient selection, meticulous bonding protocols, and comprehensive occlusal management. Overall, these restorations offer a predictable, minimally invasive, evidence-supported alternative to implants, integrating biologic preservation, clinical efficiency, and excellent esthetic and functional performance.
